# Using Video Technology to Increase Treatment Completion for Patients With Latent Tuberculosis Infection on 3-Month Isoniazid and Rifapentine: An Implementation Study

**DOI:** 10.2196/jmir.9825

**Published:** 2018-11-20

**Authors:** Chee Kin Lam, Kara McGinnis Pilote, Ashraful Haque, Joseph Burzynski, Christine Chuck, Michelle Macaraig

**Affiliations:** 1 Centers for Disease Control and Prevention Atlanta, GA United States; 2 Bureau of Tuberculosis Control New York City Department of Health and Mental Hygiene Long Island City, NY United States

**Keywords:** computer-assisted therapy, directly observed therapy, mobile phone, telemedicine, videoconferencing

## Abstract

**Background:**

Since January 2013, the New York City (NYC) Health Department Tuberculosis (TB) Program has offered persons diagnosed with latent TB infection (LTBI) the 3-month, once-weekly isoniazid and rifapentine (3HP) treatment regimen. Patients on this treatment are monitored in-person under directly observed therapy (DOT). To address patient and provider barriers to in-person DOT, we piloted the use of a videoconferencing software app to remotely conduct synchronous DOT (video directly observed therapy; VDOT) for patients on 3HP.

**Objective:**

The objective of our study was to evaluate the implementation of VDOT for patients on 3HP and to assess whether treatment completion for these patients increased when they were monitored using VDOT compared with that using the standard in-person DOT.

**Methods:**

Between February and October 2015, patients diagnosed with LTBI at any of the four NYC Health Department TB clinics who met eligibility criteria for treatment with 3HP under VDOT (V3HP) were followed until 16 weeks after treatment initiation, with treatment completion defined as ingestion of 11 doses within 16 weeks. Treatment completion of patients on V3HP was compared with that of patients on 3HP under clinic-based, in-person DOT who were part of a prior study in 2013. Furthermore, outcomes of video sessions with V3HP patients were collected and analyzed.

**Results:**

During the study period, 70% (50/71) of eligible patients were placed on V3HP. Treatment completion among V3HP patients was 88% (44/50) compared with 64.9% (196/302) among 3HP patients on clinic DOT (*P*<.001). A total of 360 video sessions were conducted for V3HP patients with a median of 8 (range: 1-11) sessions per patient and a median time of 4 (range: 1-59) minutes per session. Adherence issues (eg, >15 minutes late) during video sessions occurred 104 times. No major side effects were reported by V3HP patients.

**Conclusions:**

The NYC TB program observed higher treatment completion with VDOT than that previously seen with clinic DOT among patients on 3HP. Expanding the use of VDOT may improve treatment completion and corresponding outcomes for patients with LTBI.

## Introduction

In 2015, the World Health Organization, a leading public health organization, published an agenda that outlines the strategic direction to promote the integration of digital health concepts into tuberculosis (TB) prevention and care activities [1]. One digital health product identified that supports their strategy is the use of electronic observation of treatment [1]. In the United States, the use of video to remotely monitor patient treatment for active TB is rapidly growing [2]; however, the use of technology to monitor adherence to preventive treatment for latent TB infection (LTBI) has not been widely documented [3].

Nearly a quarter of the world’s population is infected with TB and, left untreated, many are at risk of progressing to active TB disease [4]. An important component of the US Centers for Disease Control and Prevention’s (CDC) TB elimination strategy is to expand efforts to treat individuals diagnosed with LTBI using shorter treatment regimens [5]. In 2011, CDC began recommending the use of a shorter treatment regimen, a 3-month, once-weekly regimen of isoniazid and rifapentine (3HP) under directly observed therapy (DOT), for treatment of LTBI in otherwise healthy individuals aged ≥12 years and in HIV-infected patients not taking antiretroviral medications [6,7].

In 2013, the New York City (NYC) Department of Health and Mental Hygiene (DOHMH) began offering 3HP at its TB clinics and found that treatment completion increased from a baseline of 34% with 9 months of isoniazid (9H) to 65% with 3HP [8], but it was still lower than the 82% treatment completion observed in the 3HP clinical trial [9]. Stennis et al attributed the lower than expected treatment completion to the inconvenience associated with the DOT requirement [8]. Patients in this study were treated with 3HP under in-person clinic DOT. Furthermore, among patients who chose a non-3HP treatment, 96% reported the clinic DOT requirement and 77% reported concerns about taking time away from work, child care, or other responsibilities for clinic visits as reasons they did not choose the 3HP regimen [8].

In the United States, DOT is the standard of care for monitoring patients on treatment for active TB disease, particularly those who are infectious, to ensure adherence to medication [10]. DOT requires substantial public health resources and generally is not the standard of care for patients on treatment for LTBI, a noninfectious form of TB. DOT involves trained workers observing patients ingest each dose of medication throughout the duration of treatment. DOT requires patients to either go to a clinic or have DOT workers visit patients’ homes or other locations to observe medication ingestion [11]; this can be inconvenient and disruptive for patients [12,13]. Several TB programs have explored the use of videoconferencing to remotely monitor patients on treatment for active TB, known as video DOT (VDOT). These programs have reported better or equal rates of treatment completion compared with those with in-person DOT while providing a more convenient and flexible option for patients [2,12,14-16]. VDOT uses videoconferencing software to allow patients and staff to communicate remotely via smartphones, tablets, or desktop computers. An NYC study found VDOT to be a feasible alternative to in-person DOT while improving the treatment adherence and maximizing health department resources [12]. However, to date, only one published instance known to the authors has reported using VDOT to monitor patients on treatment for LTBI [3].

To improve treatment completion for patients on 3HP, the NYC DOHMH piloted the use of live-videoconferencing technology to conduct weekly DOT observations for patients on 3HP (V3HP). The intent of the V3HP pilot was to alleviate barriers to DOT to improve treatment completion among patients started on the 3HP regimen. The objectives of this evaluation were as follows: (1) to determine the feasibility of using VDOT on patients prescribed 3HP and assess resources required to implement; (2) to compare treatment completion of patients in the V3HP pilot with previously measured 3HP treatment data; and (3) to describe challenges encountered during the pilot implementation.

## Methods

### Integration of Video Directly Observed Therapy for Treatment of Latent Tuberculosis Infection

For the V3HP implementation study, NYC DOHMH adapted the existing videoconferencing software, educational and enrollment materials, and protocols used in the previous NYC 3HP and VDOT pilot experiences [8,12]. Clinic staff received in-service training and job aids for assessing patient eligibility and referring patients to the V3HP pilot. Three nonclinical staff were trained to perform observations for V3HP, even though one performed nearly all of the observations. In addition, staff were trained in the installation and operation of the software and basic troubleshooting. Furthermore, staff were trained in documentation procedures for monitoring patients in the implementation study.

### Study Population

Eligible patients treated for LTBI with 3HP between February and October 2015 at any of the four NYC Health Department TB clinics and who met NYC DOHMH eligibility requirements for VDOT [12] were offered participation in V3HP. The diagnosis of LTBI and the prescription of 3HP were left to the discretion of providers. The eligibility for V3HP included the possession of a smartphone, tablet, or computer with videoconferencing capability; patients’ willingness to use their personal devices for VDOT sessions; access to a reliable internet connection; and agreement to a VDOT schedule. Participants were followed through the completion of treatment or up to 16 weeks from treatment initiation, whichever came first. Eligible minors were enrolled at the provider’s discretion if parental consent was obtained. Patients and guardians of minors signed a DOT agreement, which included the use of videoconferencing for observation sessions and acknowledgment of personal responsibility for costs incurred due to the use of personal devices and data service. Patients ineligible for or refused V3HP were still able to be treated with 3HP with in-person clinic DOT at any of the Health Department TB clinics but were excluded from the implementation study.

V3HP patients were prescribed medication, as per CDC guidelines [7], monthly at one of the four Health Department TB clinics. Patients returned to the clinic for monthly follow-up evaluation and medication refills. During these monthly visits, patients had the option of taking their medications in-person in lieu of their weekly VDOT sessions.

### Process Conducting Video Sessions

3HP patients were assigned to a VDOT worker who contacted the patients to verify enrollment eligibility, schedule weekly video observation sessions, remotely assist the patients in installing the Health Department-approved videoconferencing software, and test the stability of the internet connection similar to the process in a prior NYC study [12]. During each observation session, the VDOT worker logged into the videoconference at the scheduled time using a conference identifier unique to each patient and waited for the patient to log in. Observation sessions were conducted using NYC’s standard VDOT practice [12], which includes a VDOT worker asking patients at the beginning of each session if they experienced any side effects since their previous dose, and if no side effects were reported, patients were observed ingesting all prescribed medications. Patients reporting or experiencing any side effects during VDOT sessions were asked to return to the clinic or were contacted by a provider to determine the course of action. Patients were observed through the completion of therapy. No additional follow-up was performed after treatment completion or 16 weeks after treatment initiation.

Patients who failed to log in within 5 minutes of their scheduled appointment were contacted by the VDOT worker via telephone. If patients could not be reached within 30 minutes of the appointment time, a voicemail or short message service (SMS) text message was left requesting the patient to call the worker to reschedule. In addition, SMS text messaging was used to remind patients of their appointment but was used only after obtaining patient approval in accordance with the NYC DOHMH policy. The VDOT worker would attempt to call patients the following day if they had not returned the original phone call. Treatment outcomes, issues with completing VDOT observations, and other evaluation variables were documented in a V3HP database by the VDOT worker following all successful and failed VDOT sessions.

### Data Collection

Patient demographics, treatment outcomes (ie, treatment completion), and information on monthly clinic visits and clinic DOT were obtained from the TB clinic’s electronic medical record system. Treatment outcomes were categorized as follows: treatment completion (ie, completion of treatment using 3HP on VDOT), lost (ie, unable to locate after treatment initiation), refused treatment, switched treatment types, discontinued due to side effects per physician advice, and other (eg, moved). Duration of the VDOT observation sessions, outcomes of the sessions, issues encountered during sessions, and other comments pertinent to therapy sessions or failed attempts to contact patients were obtained from the V3HP database. Issues encountered during the sessions were captured as predefined codes and free text by the VDOT staff.

### Definitions

Patients were considered to have completed treatment successfully if they received at least 11 doses of 3HP within 16 weeks of treatment initiation. Issues were categorized into adherence, medical, and technical. Adherence issues consisted of 4 subcategories as follows: patient lateness (defined as >15 minutes late to a scheduled session); patient lateness for more than a day; missing or lost medications; and unapproved self-administered doses. Medical issues were included if patients reported side effects to a VDOT worker or other clinical staff or if they were documented in the electronic medical record. Technical issues were subdivided into the following 3 categories: DOHMH error, including health department computer or phone connection errors, videoconference software crashes, and audio or visual hardware malfunctions; patient equipment error, including connection difficulties, software errors, and hardware errors; and patient knowledge, including inability to operate phone or software and misunderstanding of observation requirements for VDOT.

### Analysis

The characteristics and treatment outcomes of patients in the V3HP implementation study were compared with those of 3HP patients on in-person clinic DOT who were part of an earlier NYC study implemented from January to November 2013 [8]. Participants for both studies were enrolled at NYC Health Department clinics and were included if they met the following criteria: patients being treated for LTBI; those aged ≥12 years; males or nonpregnant, nonnursing females; HIV-uninfected or -infected individuals who were not on highly active antiretroviral medications, and patients who could be contacted via telephone in case of a missed DOT visit. The significant differences in demographics and treatment outcomes between patients on 3HP with VDOT and those on clinic DOT were calculated using Pearson’s chi-square or Fisher’s exact test for categorical variables and Wilcoxon rank-sum test for continuous variables.

This implementation study was considered a public health program evaluation activity, not research, and, therefore, it did not meet the criteria to undergo review by the NYC DOHMH Institutional Review Board. Furthermore, this project was reviewed and approved at the CDC as program evaluation activity.

## Results

### Treatment Outcomes

**Figure 1:**
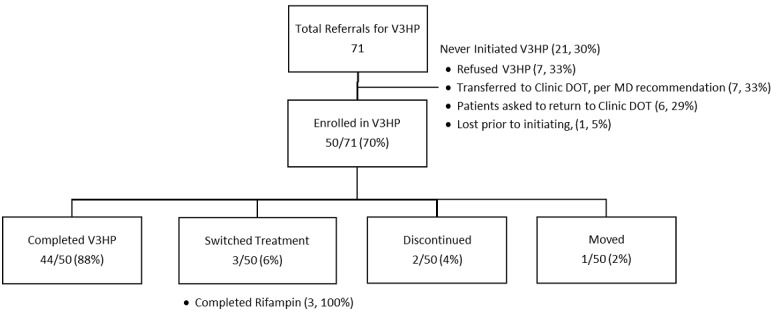
Outcomes of patients on 3-month, once-weekly treatment with isoniazid and rifapentine referred for video directly observed therapy (DOT; V3HP). MD: medical doctor.

From February to October 2015, 70% (50/71) of patients who initially agreed to V3HP were placed on VDOT. Among the V3HP patients, 88% (44/50) completed their treatment on 3HP under VDOT (Figure 1); 6% (3/50) of the additional patients completed treatment after switching to a non-3HP treatment regimen following 1-2 VDOT sessions. Of 3 patients who did not complete treatment, 2 patients opted to discontinue the treatment after experiencing headache and dizziness, respectively; 1 patient moved out of the jurisdiction after completing a single VDOT session and was referred for follow-up in the other jurisdiction. Furthermore, 21 patients who initially agreed to V3HP subsequently did not start on V3HP for various reasons (Figure 1).

There were few differences in patient demographics between V3HP patients and patients in the prior NYC 3HP study who were monitored under clinic DOT, although a higher proportion of V3HP patients were recently exposed to an infectious TB patient (Table 1). Treatment completion for V3HP patients was higher than that for 3HP patients on clinic DOT (44/50, 88%, vs 196/302, 64.9%; *P*<.001) [8].

### Video Directly Observed Therapy Sessions

Of 549 3HP treatment doses ingested by 50 V3HP patients, 65.6% (360/549) were observed under VDOT, 30.4% (167/549) doses were observed in the clinic by staff, and 4.0% (22/549) were self-administered. Patients had a median of 8 VDOT (range: 1-11) sessions. In addition, 42 patients completed 3HP treatment with 12 doses of medication and 2 patients received physician approval to discontinue therapy after 11 doses. Session times were captured for 95.8% (345/360) of VDOT sessions. The median session time was 5 (range: 1-59) minutes.

**Table 1:** Characteristics and treatment outcomes of patients on 3-month, once-weekly treatment with isoniazid and rifapentine (3HP) on clinic directly observed therapy (DOT; January to November 2013) versus video directly observed therapy (VDOT; February to October 2015) in New York City.

Characteristic		3HP Clinic DOT (n=302)		3HP VDOT (n=50)		*P* value^a^	
Male sex, n (%)		154 (51.0)		25 (50.0)		.90	
Age (years), median (interquartile range)		33 (22-45)		33.5 (25-46)		.51	
US born, n (%)		70 (23.2)		7 (14.0)		.15	
**HIV status, n (%)**						.33	
	Positive		1 (0.3)		0 (0.0)		
	Negative		161 (53.3)		32 (64.0)		
	Unknown		140 (46.4)		18 (36.0)		
**Tuberculosis risk category, n (%)**						.02^b^	
	Population Risk		187 (61.9)		27 (54.0)		
	Medical Risk		56 (18.5)		8 (16.0)		
	Contact to an active TB case		42 (13.9)		15 (30.0)		
	Other		17 (5.6)		0 (0.0)		
**Treatment outcomes, n (%)**						.001^b^	
	Completed Treatment		196 (64.9)		44 (88.0)		
	Did Not Complete		106 (35.1)		6 (12.0)		

^a^*P* value calculated using the Pearson’s chi-square or Fisher’s exact test for categorical variables and Wilcoxon rank-sum test for continuous variables.

^b^Significance at *P*<0.05.

**Table 2:** Issues encountered during the implementation study (n=205).

Issue type		Value, n (%)
**Adherence**		104 (50.7)
	Unapproved self-administer	12 (11.5)
	Patient misplaced or forgot meds	5 (4.8)
	Patient late >1 day	15 (14.4)
	Patient late >15 minutes	72 (69.2)
**Technical**		75 (36.6)
	Health department related	29 (38.7)
	Patient equipment	43 (57.3)
	Patient knowledge	3 (4.0)
Medical		26 (12.7)

**Justifications for self-administered doses of 3-month, once-weekly isoniazid and rifapentine (3HP) regimen (n=22).**

Unapproved self-administered justifications (n=12)

- Unable to reach patient. Self-administered prior to the callback: 6.
- The patient was to be observed in the clinic. The patient self-administered instead: 2.
- Technical issue. The patient self-administered rather than awaiting troubleshooting: 2.
- Administered prior to initial contact by the pilot staff: 1.
- The patient went on vacation without prior notice and did not have a video-enabled device: 1.

Approved self-administered justification (n=10)

- Administrative or holiday closure of office. Unable to schedule alternate time with the patient: 3.
- Technical issue. The patient thought observation was underway, but the video was nonfunctional: 3.
- Physician excused absence
  - Patient overseas and unable to connect: 3
  - Patient away on a meditation: 1

A total of 205 issues were encountered during the V3HP pilot among 47 patients (Table 2); 76.6% (157/205) occurred during 149 unique VDOT sessions. The remaining issues were side effects reported in-person during monthly clinic follow-ups, problems resulting in clinic DOT visits, and instances where patients self-administered medication was not under observation. Of all the issues, 50.7% (104/205) were related to patient adherence, including 12 instances where patients self-administered treatment without prior physician or program staff approval (Textbox 1). There were 26 medical issues, most of which were reported within the first 6 doses of medication (n=20). Of 37.1% (76/205) technical issues identified, a majority resulted from patient equipment errors (n=43). Health Department-related equipment errors (n=29) typically occurred in the beginning of the pilot and earlier in patients’ treatment course.

## Discussion

### Principal Considerations

This implementation study examined over 300 VDOT sessions among 50 patients on 3HP. Our analysis found that the 3HP treatment completion for patients in the implementation study increased compared with that in a prior NYC study that offered 3HP with clinic DOT (196/302, 64.9%, vs 44/50, 88%). Our evaluation supports the inclusion of VDOT to improve the completion of therapy with 3HP. Although patient nonadherence was prominent during the pilot period, with nearly half of the scheduled VDOT sessions having some form of adherence issue, the implementation study still demonstrated that staffing needs were minimal to account for the variable rescheduling time for monitoring nonadherent patients and providing reminders calls and SMS text messages when patients were late. In this implementation study, a single VDOT worker managed all observation sessions for 50 patients. Furthermore, technical issues did not prohibit the continuation of the observation sessions and the completion of treatment. This suggests that VDOT can successfully monitor patients on 3HP using minimal health department resources while offering an effective alternative for treating LTBI that removes some of the barriers to treatment completion.

In spite of these circumstances, the occurrence of patients self-administering doses remained low. A variety of causes resulted in self-administered doses, including nearly half that were approved absence by a physician or NYC staff (Textbox 1). The occurrence of self-administered doses is anticipated, and the minimal unexcused absence adds to its acceptability as an option for patient-centered care.

A recent clinical trial by Belknap et al found that in the United States, 3HP under self-administration was noninferior to 3HP under DOT [17]; however, further evaluation is needed under program settings. Therefore, the wider use of VDOT for monitoring patients on 3HP may contribute toward efforts to more rapidly reduce TB in the United States by increasing treatment completion and preventing disease.

### Strengths and Limitations

This V3HP implementation study was successfully implemented in NYC by integrating two existing programs—VDOT for monitoring patients on treatment for active TB and the 3HP short-course treatment regimen, recommended to be administered with DOT [8,12]. Staff experienced with the two initiatives were consulted to inform the implementation plan, and the few technological issues encountered were easily resolved because staff could quickly identify and address problems. The V3HP pilot required one staff person working part-time to conduct the VDOT sessions for all 50 patients enrolled during the 8-month pilot period. Furthermore, we found that patients were willing to use their own phones for VDOT sessions, a potential cost saving for health departments. However, additional evaluations including cost-effectiveness analyses comparing VDOT with in-person DOT or self-administered treatment would help quantify the value to TB programs.

This pilot also had a number of limitations. One limitation was the use of data from a prior study as the comparison group. While there were few differences between the study population for the V3HP pilot and the previous 3HP study, the V3HP pilot did enroll a greater proportion of high-risk patients with recent contact to someone with active TB disease; these high-risk individuals may have been more motivated to adhere to treatment and could have impacted treatment completion. In addition, the 2-year time difference between the previous study and the current V3HP pilot may have given clinicians an increased level of comfort in offering 3HP and less likely to discontinue the treatment because of mild or anticipated side effects. No other programmatic changes were identified between the two time periods that could have impacted the clinic population and influenced treatment completion among V3HP patients.

Enrollment in the program also presented several limitations. First, patients had to be available for VDOT sessions during limited business hours (ie, between 8:00 AM and 4:30 PM), so patients who preferred to take their medications in the early morning, late evening, or weekend could not participate. Patients opting for clinic DOT had a wider window of time to receive their weekly dose because several NYC Health Department TB clinics offer weekend and evening hours. Use of asynchronous video technology (technology that can record and timestamp video) could alleviate the scheduling constraint of live-video DOT [18]. Second, not only did patients have to use their own videoconferencing-enabled devices, but their devices also had to be compatible with the videoconferencing software approved by the DOHMH, and they were required to demonstrate the ability to use the software. Patients having a device may represent a population more inclined to accept treatment and complete through the use of video monitoring. Providing additional software options or loaner devices may make the supervision of treatment more convenient for patients and reach a wider population. Finally, patients still had to travel to the clinic for monthly follow-up visits to receive their medication. Aside from being a deterrent to patients who may not want to have to return to the clinic to receive medication, this may also have had a positive effect on treatment completion. However, as participants were able to receive medications in-person during those visits, this decreases the number of opportunities for VDOT observations. It likely accounted for the median of 8 DOT sessions per participant; the remaining observations were likely performed in-person. Further evaluation would be necessary to assess treatment completion when the number of clinic visits is reduced.

Additionally, as eligibility for and offering of 3HP is not collected as part of the program practice, the data were not available for analysis; this limits our ability to quantify the acceptance of 3HP and whether VDOT potentially increased its acceptance among patients. While there was a high proportion (21/71, 30%) of patients who did not start on V3HP, it is uncertain how these missing data affect the treatment outcome. Thus, additional studies are needed to assess DOT monitoring preferences for LTBI.

Concurrent to the pilot, a policy change in the NYC TB clinics preferentially offered another short-course treatment regimen, 4 months of daily rifampin, as an alternative to 3HP for the treatment of LTBI, which could have impacted the enrollment into the pilot. Furthermore, a shortage of rifapentine interrupted providers from offering 3HP for approximately 2 months. The overall impact of this shortage is difficult to determine because the shortage was initially not reported, but clinicians may have been aware and restricted their offering of the regimen. These events prevented the analysis to determine whether the treatment initiation with 3HP increased with VDOT. However, in taking a patient-centered approach to care, several treatment options may give patients alternatives to achieve better outcomes. Additional analysis is needed to determine whether preferentially offering of multiple short-course treatment regimens increases treatment initiation for LTBI as well as treatment completion.

### Conclusions

This evaluation shows that the use of VDOT with 3HP for the treatment of LTBI is feasible and could be integrated into the current NYC LTBI treatment practice with minimal disruption to staff time and training. Treatment completion of patients on 3HP for LTBI increased with the use of VDOT. VDOT addressed some of the barriers to in-person DOT for patients with LTBI. Programs looking to implement 3HP for the treatment of LTBI should consider evaluating the use of VDOT. Further research is necessary to assess the use of VDOT for patients on treatment with 3HP compared with self-administration in a programmatic setting. Additionally, it may be worth exploring the expansion of the use of asynchronous videoconferencing technology, thereby further reducing the intrusiveness of DOT.

## Abbreviations

3HP: 3-month, once-weekly isoniazid and rifapentine regimen
9H: 9-month daily isoniazid regimen
CDC: United States Centers for Disease Control and Prevention
DOHMH: Department of Health and Mental Hygiene
DOT: directly observed therapy
LTBI: latent tuberculosis infection
NYC: New York City
SMS: short message service
TB: tuberculosis
VDOT: video directly observed therapy
V3HP: video directly observed therapy for patients on a 3-month, once-weekly isoniazid and rifapentine regimen
